# The influence of experimentally induced polyploidy on the relationships between endopolyploidy and plant function in *Arabidopsis thaliana*

**DOI:** 10.1002/ece3.5886

**Published:** 2019-12-18

**Authors:** Evan K. Pacey, Hafiz Maherali, Brian C. Husband

**Affiliations:** ^1^ Department of Integrative Biology University of Guelph Guelph ON Canada

**Keywords:** *Arabidopsis thaliana*, colchicine, endopolyploidy, induced polyploidy, phenotype, polyploidy, trade‐off, whole genome duplication

## Abstract

Whole genome duplication, leading to polyploidy and endopolyploidy, occurs in all domains and kingdoms and is especially prevalent in vascular plants. Both polyploidy and endopolyploidy increase cell size, but it is unclear whether both processes have similar effects on plant morphology and function, or whether polyploidy influences the magnitude of endopolyploidy. To address these gaps in knowledge, fifty‐five geographically separated diploid accessions of *Arabidopsis thaliana* that span a gradient of endopolyploidy were experimentally manipulated to induce polyploidy. Both the diploids and artificially induced tetraploids were grown in a common greenhouse environment and evaluated with respect to nine reproductive and vegetative characteristics. Induced polyploidy decreased leaf endopolyploidy and stem endopolyploidy along with specific leaf area and stem height, but increased days to bolting, leaf size, leaf dry mass, and leaf water content. Phenotypic responses to induced polyploidy varied significantly among accessions but this did not affect the relationship between phenotypic traits and endopolyploidy. Our results provide experimental support for a trade‐off between induced polyploidy and endopolyploidy, which caused induced polyploids to have lower endopolyploidy than diploids. Though polyploidy did not influence the relationship between endopolyploidy and plant traits, phenotypic responses to experimental genome duplication could not be easily predicted because of strong cytotype by accession interactions.

## Graphical abstract

Colchicine‐induced polyploidy lowered endopolyploidy in 55 natural accessions of *Arabidopsis thaliana*. Accessions varied in phenotypic responses to induced polyploidy but this did not affect the relationship between phenotypic traits and endopolyploidy.

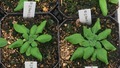

## 1 INTRODUCTION

Whole genome duplication (WGD), the increase in whole chromosome sets, is widespread throughout the tree of life. Numerous cases of WGD have been documented in bacteria, archaea, fungi, and animals (Albertin & Marullo, 2012; Nagl, 1976; Oliverio & Katz, 2014; Otto & Whitton, 2000). However, the highest incidence of WGD exists in vascular plants (Barow, 2006; Cui et al., 2006; Jiao et al., 2011; Nagl, 1976; Otto & Whitton, 2000). This prevalence of WGD in plants makes them an opportune study system for investigating the functional significance of WGD and its effects on phenotype and fitness.

One of the most common effects of WGD is that it often increases cell size (del Pozo & Ramirez‐Parra, 2015; Melaragno, Mehrotra, & Coleman, 1993; Otto & Whitton, 2000; te Beest et al., 2012). This effect causes the surface area:volume ratio of a cell to decrease which can have consequences at the tissue and whole‐organism level (Bennett, 1971, 1972; Levin, 1983; Otto & Whitton, 2000). For example, tissue composed of larger cells with low surface area:volume ratios should have higher net volume of intracellular storage space and lower net volume of cell wall than an equal volume of tissue comprised of smaller cells. This trade‐off between intracellular storage and cellular wall could affect a tissue or organism in many ways, altering an organism's function and response to environment. For example, WGD might be favoured in environments characterized by drought, where enhanced water storage capacity relative to biomass investment could facilitate survival through periods of water limitation (De Rocher, Harkins, Galbraith, & Bohnert, 1990; Schwinning & Ehleringer, 2001). However, such an adaptation would come at the expense of greater diffusion barriers to gas exchange, which could limit photosynthesis and offset increased water storage with reduced water use efficiency while also causing weakened structural support (Corneillie et al., 2019; Niklas, 1992, 1994).

Whole genome duplication that affects every cell in an organism (including gametes) is called polyploidy. Polyploidy can result from duplication of genomes in an interspecific hybrid (allopolyploidy) or of a single species (autopolyploidy) (Comai, 2005; del Pozo & Ramirez‐Parra, 2015; Levin, 1983; Otto & Whitton, 2000; Soltis, Buggs, Doyle, & Soltis, 2010; te Beest et al., 2012). Autopolyploidy is a useful system for studying the phenotypic effects of polyploidy because autopolyploidy primarily reflects changes in genome size, whereas allopolyploids exhibit impacts of hybridization as well as polyploidy (Comai, 2005; del Pozo & Ramirez‐Parra, 2015; Levin, 1983; Otto & Whitton, 2000; Soltis et al., 2010; te Beest et al., 2012). The effects of autopolyploidy on phenotype reflect the downstream impacts of increased cell size (a nucleotypic effect that is independent of the informational content of the genome; Bennett, 1971, 1972) plus any additional genetic effects of increased gene copy number, gene expression, and postduplication evolutionary change (Comai, 2005; del Pozo & Ramirez‐Parra, 2015; Levin, 1983; Otto & Whitton, 2000; Soltis et al., 2010; te Beest et al., 2012). Indeed, many of the phenotypic changes seen in polyploid plants may be caused by increased cell size relative to cell number (although a compensation effect where cell number decreases to offset increased cell size can also occur; see Hisanaga, Kawade, & Tsukaya, 2015) which could also explain why polyploid plants often exhibit larger body, organ, and leaf size than their progenitors (Comai, 2005; del Pozo & Ramirez‐Parra, 2015; Müntzing, 1936; Levin, 1983; Otto & Whitton, 2000; Soltis et al., 2010; te Beest et al., 2012).

In contrast to polyploidy, endopolyploidy refers to WGD events that occur within some somatic cells and tissues of a given individual. Similar to polyploidy, endopolyploidy often increases cell size (although this can depend on tissue type; see Katagiri et al., 2016), which may affect phenotypes in the same way as polyploidy. However, unlike polyploidy, the degree of endopolyploidy in a particular accession can vary with environmental stimuli (Jovtchev, Barow, Meister, & Schubert, 2007; Scholes & Paige, 2015). This plasticity allows endopolyploid cells and tissues to produce a range of growth‐related phenotypes in response to prevailing environmental conditions. Endopolyploidy can also be genetically determined and vary among accessions from different environments (for an excellent review of endopolyploidy in seed plants see Barow, 2006; Gegas et al., 2014; Pacey, 2018).

Comparative analyses among species suggest that a trade‐off may occur between polyploidy and endopolyploidy. For example, species with small genomes or low polyploidy are more likely to exhibit high levels of endopolyploidy, whereas species with large genomes or high polyploidy often have little or no endopolyploidy (Bainard, Bainard, Henry, Fazekas, & Newmaster, 2012; Barow & Meister, 2003; De Rocher et al., 1990; Nagl, 1976). These patterns suggest there are developmental or structural constraints on maximum cell size for species with large genomes or high polyploidy (Barow, 2006; De Rocher et al., 1990). However, these comparisons among species cannot be used to infer whether polyploidy actually causes species with large genomes to have reduced endopolyploidy. Experimental studies that manipulate polyploidy are required to determine if increased polyploidy causes a reduction in endopolyploidy.

Empirical studies of the effects of induced autopolyploidy on the expression of endopolyploidy have variable results. For example, in *Datura stramonium*, *Hyoscyamus niger* and *Portulaca grandiflora*, synthetic autopolyploids have decreased endopolyploidy compared to diploids (Mishiba & Mii, 2000; Weber, Georgiev, Pavlov, & Bley, 2008). By contrast, the degree of endopolyploidy in *A. thaliana* appears to be insensitive to induced autopolyploidy (del Pozo & Ramirez‐Parra, 2014). Jovtchev et al. (2007) observed lower endopolyploidy in natural *A. thaliana* tetraploids compared to diploids; however, the tetraploids and diploids were from different accessions, and thus polyploidy could not be determined as the cause of reduced endopolyploidy. The apparent insensitivity of *A. thaliana* endopolyploidy to experimentally induced autopolyploidy could be due to its relatively small genome size (Schmuths, Meister, Horres, & Bachmann, 2004). Alternatively, past studies have been based on only two accessions (Col‐0 and *Ler*); hence the results to date may not reflect the general effects of autopolyploidy on endopolyploidy in this species (del Pozo & Ramirez‐Parra, 2014; Rédei, 1992). It is not known if endopolyploidy in naturally occurring accessions would respond differently to induced autopolyploidy. Because *A. thaliana* is known to show extensive natural variation for the degree of endopolyploidy, it could serve as an important model system to make general inferences about how inducing autopolyploidy influences the expression of endopolyploidy (Gegas et al., 2014; Pacey, 2018).

**Figure 1:**
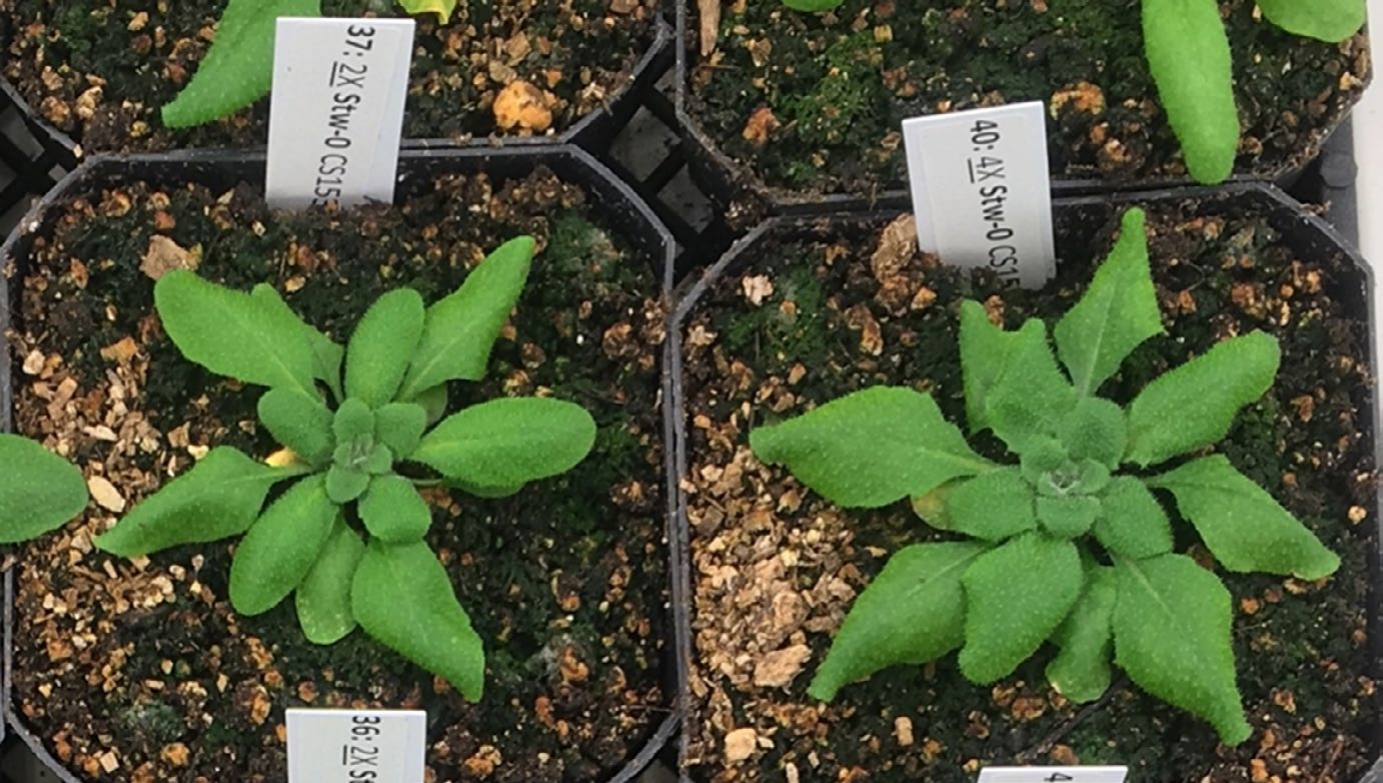
Organism photo of an experimentally induced tetraploid (4x; right) beside its natural diploid (2x; left) progenitor *Arabidopsis thaliana* accession (Stw‐0, CS1538)

To explore the relationship between induced polyploidy and endopolyploidy, we examined the degree of endopolyploidy in natural diploid and synthesized autotetraploid accessions of *A. thaliana* (Figure 1). Though *A. thaliana* has experienced at least two ancient WGD events, polyploidy has been lost over evolutionary time in this species, and nearly all wild populations function as diploids (del Pozo & Ramirez‐Parra, 2015). We used a common garden experiment to address the following two questions. Does induced polyploidy decrease endopolyploidy in natural accessions of *A. thaliana*? Does the degree of endopolyploidy affect growth and reproductive traits differently in diploids versus tetraploids? We hypothesize that induced polyploidy decreases endopolyploidy in natural accessions of *A. thaliana* because there are developmental or structural constraints on maximum cell size. We also hypothesize that the degree of endopolyploidy will not affect growth and reproductive traits differently in diploids versus tetraploids because endopolyploidy should influence cell size independently of polyploidy.

## 2 MATERIALS AND METHODS

### 2.1 Source populations and polyploid synthesis

To explore how induced polyploidy affects endopolyploidy and growth/reproductive traits in *A. thaliana*, seed families from 55 randomly selected geo‐referenced diploid accessions of *A. thaliana* that span its native geographic range (collected as part of the 1,001 genomes project and made available through the *Arabidopsis* Biological Resource Center) and are facultative in their vernalization requirement were used (Pigliucci, 2002; Weigel & Mott, 2009; Appendix Table A1). Seeds from each accession were germinated on an agarose medium, bathed in colchicine (0.5% for 1–2 hr) to induce polyploidy and then transplanted to soil (Yu, Haage, Streit, Gierl, & Torres Ruiz, 2009; A. Green & B. C. Husband, unpublished). Inflorescences on plants from colchicine‐treated seeds were checked for cytotype using flow cytometry and seeds were collected from each inflorescence separately (to prevent mixing of seeds from other inflorescences and parents) and then stored in their own individual vial (A. Green & B. C. Husband, unpublished). These seeds, which were one generation removed from the colchicine treatment, were unlikely to show unintended effects of colchicine (Münzbergová, 2017). To confirm this, a previous study compared vegetative and reproductive traits between colchicine‐treated and ‐untreated diploids and found no difference (A. Green & B. C. Husband, unpublished). To minimize this concern further, in this study, we used diploid seed that had received a colchicine treatment so any observed differences between ploidy levels were not confounded with colchicine treatment.

### 2.2 Growth conditions and experimental design

To overcome seed dormancy and synchronize germination, diploid and tetraploid seeds of each accession were stratified at 3°C in the dark for 72 hr in Parafilm^®^ enclosed petri dishes containing moist filter paper. Three germinating seeds (that came from the same inflorescence seed vial) for each accession and cytotype replicate were placed on the soil of a single 7.3 cm deep, 377 cm^3^ dark green pot (KORD Products) containing Sunshine Mix #4 (Sun Gro Horticulture). Plants were thinned to one per pot when true leaves developed (the healthiest seedling by appearance was kept). All pots were randomly placed in eight‐liter white trays, which each hold up to 18 pots (ITML Horticulture Products Inc.), watered weekly with 18‐9‐18 fertilizer mixed at a rate of 200 ppm until trays overflowed and then allowed to fully drain (which required 10 min). Trays were also watered (without fertilizer) every 2 days after day 14 postpotting until they overflowed and then allowed to fully drain (which required 10 min).

Plants were grown in a controlled environment (23°C day, 20°C night, 16 hr daylight) at the University of Guelph Phytotron greenhouse. Supplemental lighting using 600 W high‐pressure sodium light fixtures (P.L. Light Systems Inc.) with SON‐T bulbs (Philips) that delivered ~300 µmol/m^2^/s at greenhouse bench level were used when natural sunlight was inadequate (≤400 µmol/m^2^/s) during the assigned 16 hr photoperiod. Plants were grown in two groups of three randomized temporal blocks (the first group of three blocks was grown 1 week apart in November 2015 while the second group of three blocks was grown 1 week apart in June 2016 using the same methods) with each block containing one individual from each accession and cytotype. Each plant from the November 2015 group of three blocks was measured for day of bolting (when the primary stem first emerged), leaf endopolyploidy index (EI), stem EI, and stem height (single block *N* = 110, three combined blocks from November 2015 *N* = 330 with *N* = 3 for each accession and cytotype). Due to the destructive nature of flow cytometry, the second group of three temporal blocks (grown in June 2016) was needed and used to measure leaf size, leaf dry mass, specific leaf area (SLA), leaf water content, and chlorophyll concentration (single block *N* = 110, three combined blocks from June 2016 *N* = 330 with *N* = 3 for each accession and cytotype).

### 2.3 Flow cytometry and endopolyploidy index

To estimate the degree of endopolyploidy in each individual, the largest rosette leaf was harvested on the first day of bolting (when the primary stem first emerged). The primary stem from these same individuals was harvested on the first day of anthesis (or fruiting if anthesis was not clearly visible) and its length was measured. To prepare leaf tissue for flow cytometry, the right half of the leaf blade (when the abaxial epidermis was facing upwards and the petiole was pointed toward the researcher) was cut off with a razorblade without including its midrib (to reduce tissue heterogeneity). To prepare stem tissue, all petioles, cauline leaves and flowers (to reduce tissue heterogeneity) were removed. Nuclei from leaf and stem tissue were then isolated separately by finely chopping with a fresh razor blade in Galbraith's buffer (Galbraith et al., 1983). Propidium iodide (100 µg/ml) and RNAse (0.5 µg/ml) were added to the buffer to stain DNA and degrade interfering RNA, respectively. The mixture of tissue and buffer was filtered (30 µm filters; Partec GmbH) to remove tissue fragments and allowed to stain for 20–60 min.

**Figure 2:**
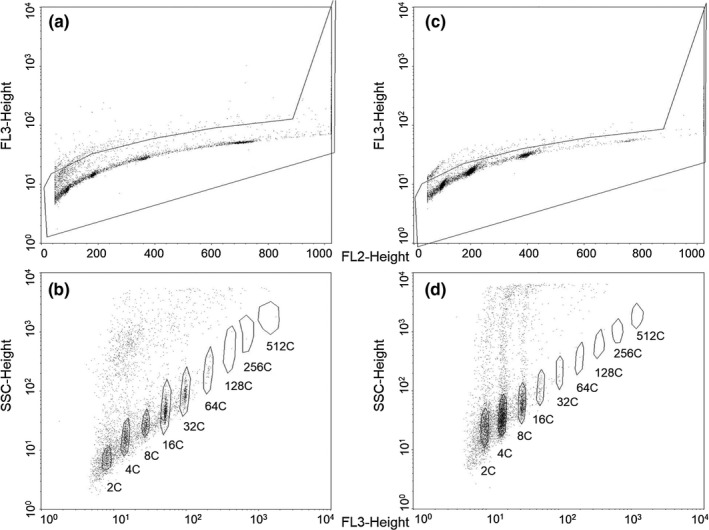
Flow cytometry manual gating examples for a single diploid (2x) *Arabidopsis thaliana* plant. (a) Leaf debris gating, (b) leaf nuclei cluster gating, (c) stem debris gating, (d) stem nuclei cluster gating. The analysis software used only allowed for nine nuclei cluster gates so 1024C and 2048C nuclei were counted manually and added to total gated events

**Figure 3:**
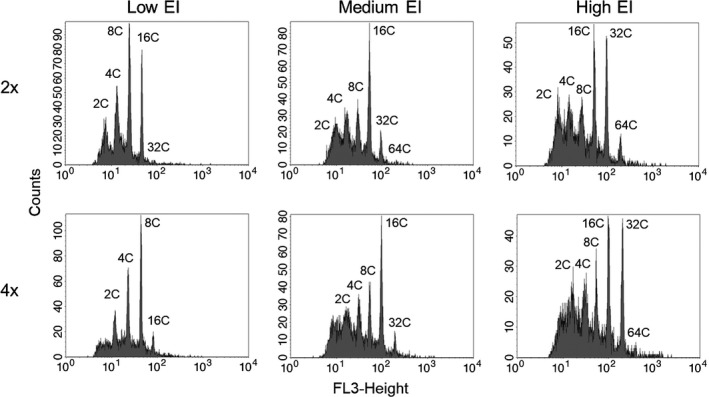
Flow cytometry histograms of low, medium and high endopolyploidy index (EI) *Arabidopsis thaliana* leaves that span diploid (2x) and tetraploid (4x) cytotypes. *X*‐axes represent log FL3‐Height (670 nm) fluorescence while *Y*‐axes represent the number of individual nuclei detected for each ploidy level (2C, 4C, 8C etc). Note that the 2C peak of a diploid is 2x (4C = 4x, 8C = 8x etc.) while the 2C peak of a tetraploid is 4x (4C = 8x, 8C = 16x etc.)

DNA content of individual nuclei was measured with flow cytometry using a FACSCalibur flow cytometer and CellQuest Pro software (BD Biosciences). Debris was minimized by gating using a FL2‐height (585 nm) versus FL3‐height (670 nm) scatterplot (Figure 2a,c), while nuclei clusters (2C, 4C, 8C etc.) were gated using a log FL3‐height (670 nm) versus side scatter scatterplot (Figure 2b,d). Nuclei peak means were measured on a log FL3‐height (670 nm) histogram (Figure 3). The positions and sizes of gates required only minor adjustments between samples and tissues. The 2C peak was prominent and readily detected in stem tissue, and thus was used as a marker to confirm the location of the 2C peak in leaf tissue for each accession. At least 5,000 nuclei were counted for leaf and stem samples from each individual plant and the ploidy identified from their fluorescence peaks (representing 2C, 4C, 8C etc. values and ranging from 2C to 2,048C as indicated by their respective flow cytometry histograms; Figure 3). The extent of endopolyploidy in each tissue was then calculated using the cycle value or Endopolyploidy Index (EI) equation (Barow & Meister, 2003):

$$\text{EI}=\frac{\left(0*n_{2C}+1*n_{4C}+2*n_{8C}+3*n_{16C}\ldots \right)}{\left(n_{2C}+n_{4C}+n_{8C}+n_{16C}\ldots \right)}$$

where *n*_#c_ refers to the number of nuclei of the # C value and where # ranges from 2, 4, 8, 16 to the maximum detected. Note that EI is a single value measure of the number of rounds of endopolyploidy in a sample over its base genome size (2C). The 2C peak of a diploid is 2x (C = amount of DNA within an unreplicated gametic genome, x = number of sets of chromosomes) while the 2C peak of a tetraploid is 4x (Figure 3). Aside from this difference (which is not included in the calculation), the EI calculation is the same whether it is a diploid or tetraploid plant. Therefore, the lack of a diploid measure in tetraploids does not affect the EI calculation and makes their EI comparable to the EI of a diploid.

The EI was used over other measures of endopolyploidy due to its simplicity. Reporting nuclei number for each ploidy level (2C, 4C, 8C etc.) provides more detailed information on the distribution of ploidy; however, it is more cumbersome, statistically, to evaluate variation in endopolyploidy (Gegas et al., 2014). Using the mean C‐value of an individual sample provides a single continuous value; however, it places an overemphasis on higher ploidy levels because of the exponential nature of increasing ploidy (Barow & Meister, 2003). Note that if two samples with different combinations of endopolyploidy gave the same EI, they were considered to be equivalent in their degree of endopolyploidy regardless of whether they were functionally equivalent.

### 2.4 Plant functional traits

To compare the relationships between endopolyploidy and plant functional traits between diploids and induced tetraploids, we measured days to bolting, leaf EI, stem EI, and stem height in the first set of temporal blocks. The second set of temporal blocks was used to measure leaf size, leaf dry mass, leaf water content, leaf chlorophyll concentration, and SLA on the largest rosette leaf on the first day of bolting. Specific leaf area was included because it is considered a key plant functional trait and has been hypothesized to be higher in leaves with larger cells than equally sized leaves with smaller cells because larger‐celled leaves should have relatively less cellular wall mass due to their change in cellular surface area:volume ratio (Shipley, Lechowicz, Wright, & Reich, 2006; Wright et al., 2004).

Apparent chlorophyll content was taken as the average of SPAD meter (SPAD‐502, Minolta Camera Co. Ltd.) measurements at the base, middle, and tip of the leaf (Ling, Huang, & Jarvis, 2011). Leaf size (cm^2^) was measured using a leaf area meter (LI‐3100, LI‐COR Inc.). Leaves were then weighed, dried in a drying oven at 60°C for 48 hr, and then reweighed to obtain dry mass (g). Leaf water content (g) was calculated as the wet mass minus the dry mass divided by the dry mass. Specific leaf area (cm^2^/g) was calculated as the leaf area divided by dry mass.

### 2.5 Statistical analyses

To test the hypothesis that induced polyploidy decreases endopolyploidy in natural accessions of *A. thaliana*, we used a two‐way ANOVA of individual plant values with accession, cytotype, accession * cytotype, and block as fixed factors. The coefficient of variation (CV) for traits was calculated by dividing their standard deviation by their mean. To test the hypothesis that the regression slope between endopolyploidy and each growth/reproductive trait will be equal for diploids and tetraploids, we used an ANCOVA on accession means with cytotype as a fixed factor and EI as a covariate. We tested for homogeneity of slopes by looking at the interaction between cytotype and EI, where a nonsignificant effect means that the slopes are not statistically different. Leaf dry mass, leaf size, chlorophyll concentration, and days to bolting violated the ANOVA and ANCOVA assumptions of normality and equality of variances and hence were log_10_ transformed. All statistical analyses were completed with SPSS 24 (IBM) and graphed with Sigma Plot 12.5 (Systat software Inc.).

## 3 RESULTS

**Figure 4:**
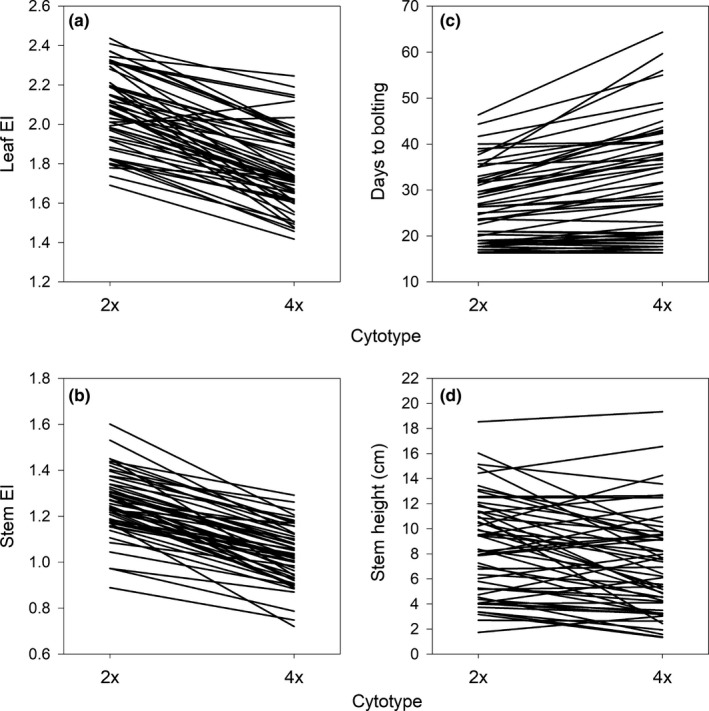
Reaction norms showing average leaf, stem and reproductive traits for diploid (2x) and tetraploid (4x) forms of each *Arabidopsis thaliana* accession. (a) Leaf endopolyploidy index (EI), (b) stem EI, (c) days to bolting and (d) stem height

**Figure 5:**
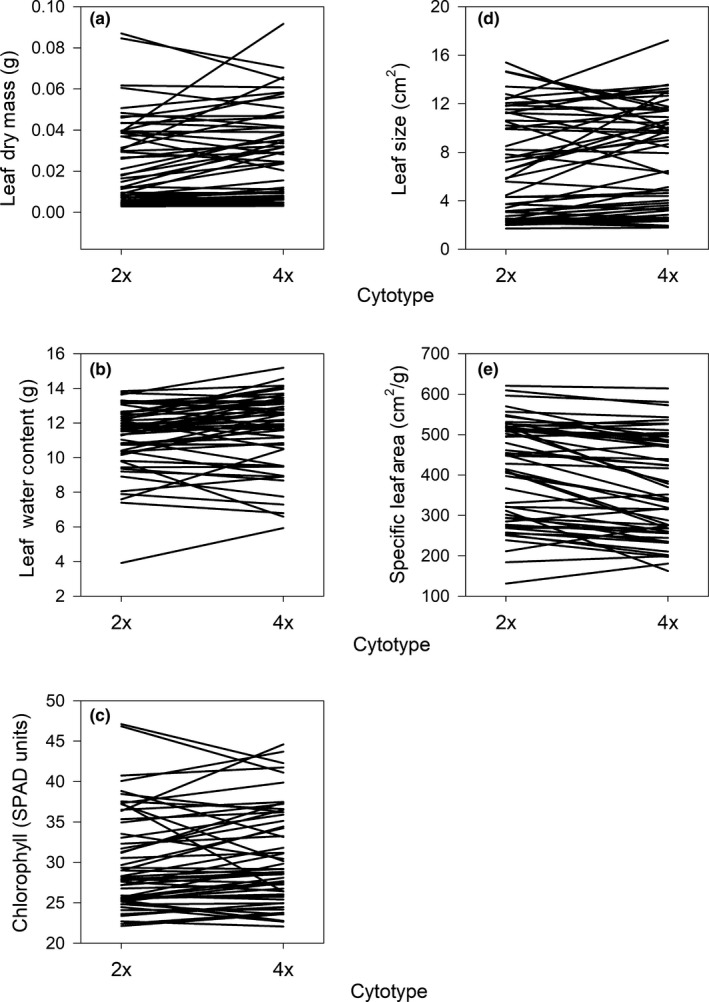
Reaction norms showing average leaf traits for diploid (2x) and tetraploid (4x) forms of each *Arabidopsis thaliana* accession. (a) Leaf dry mass, (b) water content, (c) chlorophyll concentration, (d) leaf size and (e) specific leaf area

Accessions of *A. thaliana* differed significantly for all nine traits (Figures 4 and 5, Table 1). Leaf dry mass (CV = 0.934) was the most variable followed by leaf size (CV = 0.622), stem height (CV = 0.574), days to bolting (CV = 0.384), SLA (CV = 0.331), and leaf chlorophyll content (CV = 0.219) (Figures 4 and 5, Table 1). The three traits that displayed the least amount of variation among accessions were leaf water content (CV = 0.203), stem EI (CV = 0.167), and leaf EI (CV = 0.153) (Figures 4 and 5, Table 1).

**Table 1:** Two‐way analysis of variance for accession, cytotype, accession * cytotype and block for nine phenotypic traits in *Arabidopsis thaliana*

Trait	Term			
	Accession	Cytotype	Accession * Cyto	Block
Leaf EI				
MS	0.193	7.633	0.032	0.981
*F*	6.916	273.552	1.159	35.161
*p*	<.001	<.001	.231	<.001
Leaf dry mass (g)				
MS	1.099	0.717	0.040	0.203
*F*	55.662	36.284	2.002	10.296
*p*	<.001	<.001	<.001	<.001
Leaf water content (g)				
MS	22.092	9.645	1.973	2.451
*F*	11.478	5.011	1.025	1.274
*p*	<.001	.026	.438	.282
Leaf size (cm^2^)				
MS	0.518	0.208	0.023	0.228
*F*	40.669	16.371	1.803	17.916
*p*	<.001	<.001	.002	<.001
SLA (cm^2^/g)				
MS	8.717 × 10^4^	1.609 × 10^5^	3,507.191	7.834 × 10^3^
*F*	38.808	47.605	1.561	3.488
*p*	<.001	<.001	.014	.032
Chlorophyll (SPAD units)				
MS	0.037	0.004	0.003	0.009
*F*	19.120	2.062	1.362	4.563
*p*	<.001	.152	.065	.011
Bolting (Days to)				
MS	0.127	0.305	0.005	0.010
*F*	65.238	156.381	2.664	5.050
*p*	<.001	<.001	<.001	.007
Stem EI				
MS	0.082	3.961	0.013	0.227
*F*	7.996	388.021	1.307	22.218
*p*	<.001	<.001	.096	<.001
Stem height (cm)				
MS	79.585	151.287	11.026	18.584
*F*	11.037	20.981	1.529	2.577
*p*	<.001	<.001	.019	.078

Note

55 accessions and 2 cytotypes were grown in three temporal blocks for each trait, *N* = 318–324 for each trait (includes diploids and tetraploids), *N* = 2–3 for each specific accession and cytotype, *N* = 1 for 4x Sij‐1 Stem EI and Stem Height. *df* for accession term = 54, cytotype term = 1, accession * cytotype term = 54, block term = 2. Leaf EI = leaf endopolyploidy index, SLA = specific leaf area, Chlorophyll = chlorophyll concentration, Stem EI = stem endopolyploidy index. Leaf dry mass, leaf size, chlorophyll and bolting were log_10_ transformed.

Induced polyploidy significantly reduced mean leaf EI by 15%, from 2.1 in diploids to 1.8 in tetraploids (Figure 4a), and stem EI by 18%, from 1.3 in diploids to 1 in tetraploids (Figure 4b) (Table 1, Appendix Table A1; see Appendix Tables A2 and A3 for average percentage of leaf and stem nuclei within each ploidy state and greater or equal to 4C). Induced polyploidy significantly increased mean days to bolting by 19%, from 26.5 days in diploids to 31.4 days in tetraploids (Figure 4c), dry mass by 14%, from 0.02 g in diploids to 0.03 g in tetraploids (Figure 5a), water content by 4%, from 11.2 g in diploids to 11.6 g in tetraploids (Figure 5b), and leaf size by 8%, from 7 cm^2^ in diploids to 7.5 cm^2^ in tetraploids (Figure 5d) (Table 1). By contrast, induced polyploidy significantly decreased mean SLA by 8%, from 412.3 cm^2^/g in diploids to 378.3 cm^2^/g in tetraploids (Figure 5e) and stem height by 15%, from 8.6 cm in diploids to 7.3 cm in tetraploids (Figure 4d) (Table 1). Induced polyploidy did not affect mean chlorophyll concentration (Figure 5c, Table 1).

The effect of induced polyploidy on leaf and stem EI was uniform among accessions (i.e., no cytotype by accession interaction), decreasing mean values in most cases (Figure 4a,b, Table 1, Appendix Table A1). However, accessions responded differently to induced polyploidy in five other traits measured (Figures 4 and 5, Table 1). Induced polyploidy increased days to bolting by up to 71% in one accession and decreased it by as much as 4% in another (Figure 4c, Table 1). Stem height increased by up to 39% and decreased by as much as 78% (Figure 4d, Table 1), and leaf dry mass increased by up to 134% and decreased by as much as 26%, depending on accession (Figure 5a, Table 1). Induced polyploidy increased leaf size up to 125% and decreased it by 35% (Figure 5d, Table 1), and SLA increased by 30% and decreased by 29%, depending on accession (Figure 5e, Table 1).

**Figure 6:**
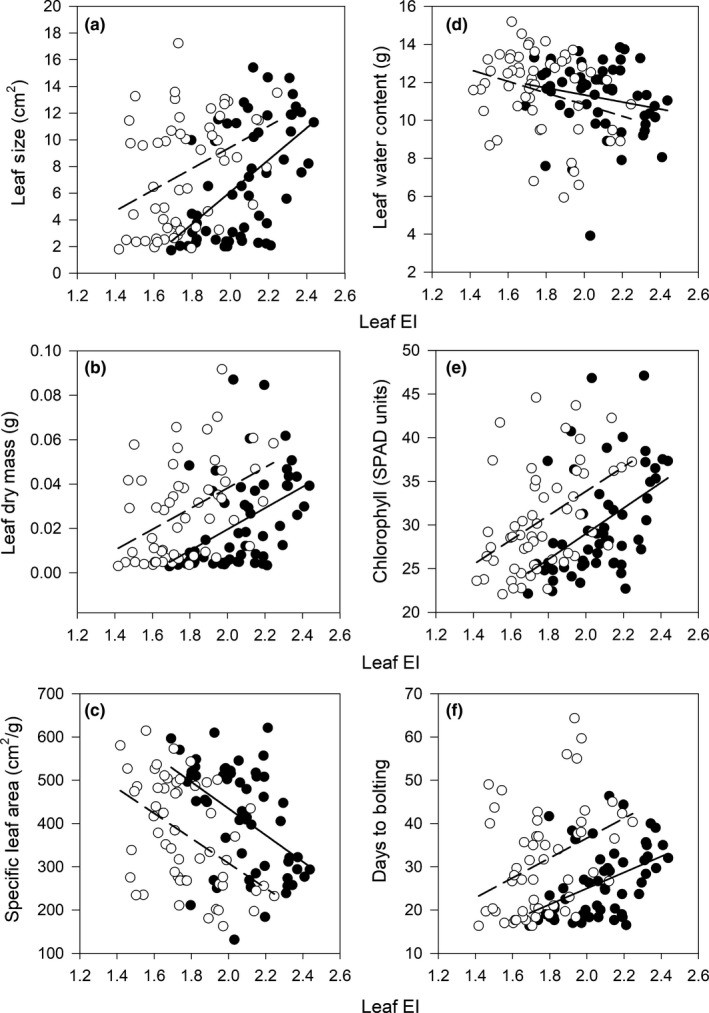
Relationships between average leaf endopolyploidy index (EI) and average plant functional traits in *Arabidopsis thaliana*. (a) Leaf size, (b) dry mass, (c) specific leaf area, (d) water content, (e) chlorophyll concentration and (f) days to bolting for 2x (closed circles) and 4x (open circles) cytotypes. Regression lines were drawn for 2x (solid line) and 4x (broken line) cytotypes

**Figure 7:**
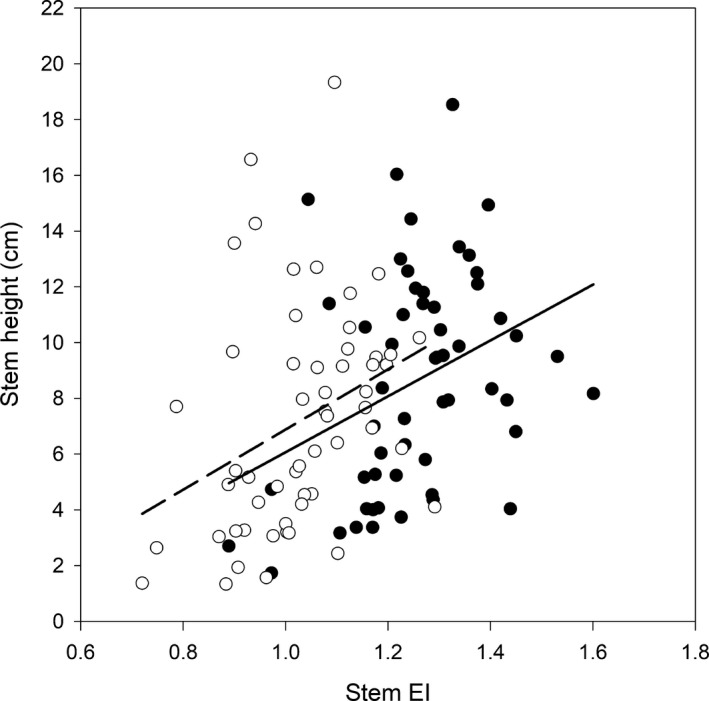
Relationship between average stem endopolyploidy index (EI) and average stem height for 2x (closed circles) and 4x (open circles) *Arabidopsis thaliana* cytotypes. Regression lines were drawn for 2x (solid line) and 4x (broken line) cytotypes

The endopolyploidy index was positively associated with leaf size (Figure 6a), dry mass (Figure 6b), chlorophyll (Figure 6e), days to bolting (Figure 6f), and stem height (Figure 7, Table 2). Endopolyploidy Index was negatively correlated with SLA (Figure 6c) and water content (Figure 6d) (Table 2). However, the slope of the relationships between EI and plant trait values did not differ between diploids and tetraploids, as indicated by the lack of a significant interaction between cytotype and EI in the ANCOVA (Figures 6 and 7, Table 2).

**Table 2:** Analysis of covariance for cytotype, EI, and cytotype * EI for seven phenotypic trait averages in *Arabidopsis thaliana*

Trait	Term		
	Cytotype	EI	Cytotype * EI
Leaf dry mass (g)			
MS	0.221	5.089	0.059
*F*	1.492	34.322	0.401
*p*	.225	<.001	.528
Leaf water content (g)			
MS	0.983	25.409	1.495
*F*	0.250	6.468	0.380
*p*	.618	.012	.539
Leaf size (cm^2^)			
MS	0.184	2.298	0.081
*F*	2.671	33.422	1.180
*p*	.105	<.001	.280
SLA (cm^2^/g)			
MS	7,575.020	3.724 × 10^5^	386.162
*F*	0.618	30.386	0.032
*p*	.434	<.001	.859
Chlorophyll (SPAD units)			
MS	0.003	0.175	<0.001
*F*	0.478	33.063	0.034
*p*	.491	<.001	.855
Bolting (Days to)			
MS	0.005	0.443	<0.001
*F*	0.255	23.327	0.012
*p*	.615	<.001	.912
Stem height (cm)			
MS	0.001	194.266	0.268
*F*	<0.001	13.676	0.019
*p*	.995	<.001	.891

Note

55 accessions and 2 cytotypes were used for each trait, *N* = 318–324 for each trait (includes diploids and tetraploids), *N* = 2–3 for each specific accession and cytotype trait average, *N* = 1 for 4x Sij‐1 Stem EI and Stem Height. *df* for cytotype, EI, and cytotype * EI terms = 1. EI = endopolyploidy index, SLA = specific leaf area, Chlorophyll = chlorophyll concentration. Leaf EI was used as a covariate for all traits except for Stem Height which used Stem EI as a covariate. Leaf dry mass, leaf size, chlorophyll and bolting were log_10_ transformed.

## 4 DISCUSSION

We found evidence for a trade‐off between induced polyploidy and endopolyploidy in natural accessions of *A. thaliana*. Our hypothesis that induced polyploidy would decrease the degree of endopolyploidy was supported; specifically, induced polyploidy lowered endopolyploidy in leaf and stem tissue by 15% and 18%, respectively (Figure 4a,b, Table 1, Appendix Table A1). Moreover, our hypothesis that the degree of endopolyploidy would not affect growth and reproductive traits differently between cytotypes was supported as the regression slopes between endopolyploidy and growth/reproductive traits did not differ between cytotypes (Figures 6 and 7, Table 2). These findings indicate that even though induced polyploidy can limit the expression of endopolyploidy, such effects do not appear to influence the relationship between endopolyploidy and plant functional traits. Thus, the trade‐off between induced polyploidy and endopolyploidy does not appear to influence the functional consequences of variation in endopolyploidy in *A. thaliana*.

The trade‐off between induced polyploidy and endopolyploidy in natural accessions of *A. thaliana* confirmed and contradicted evidence from previous studies. Our observation that induced polyploidy significantly decreased average leaf and stem endopolyploidy (Figure 4a,b, Table 1, Appendix Table A1) was in agreement with previous studies on diploid and tetraploid *D. stramonium*, *H. niger* and *P. grandiflora* (Mishiba & Mii, 2000; Weber et al., 2008). However, our results contrast with a similar, but smaller study on *A. thaliana* that found no difference in leaf endopolyploidy between X‐ray (Col‐0) and colchicine‐induced (*Ler*) autotetraploids and their diploid parental lines (del Pozo & Ramirez‐Parra, 2014). Moreover, that study found no difference in leaf size between cytotypes while our study showed that tetraploids had significantly larger (8%) leaves than diploids (del Pozo & Ramirez‐Parra, 2014; Figure 5d, Table 1). These contrasting results may be caused by differences in the accessions used in the present study compared to the Col‐0 and *Ler* accessions used by del Pozo and Ramirez‐Parra (2014). These accessions are relatively small and have undergone multiple generations of evolution in a laboratory environment and may not be representative of field populations (del Pozo & Ramirez‐Parra, 2014; Rédei, 1992). For example, the leaf sizes of Col‐0 and *Ler* ranged from 1.1 to 1.2 cm^2^ while the 55 natural accessions we used were much larger as they had leaf sizes that ranged from 1.71 to 15.41 cm^2^ (an increase of ~33%–1340%) (del Pozo & Ramirez‐Parra, 2014; Figure 5d).

Induced polyploidy did not influence the relationship between endopolyploidy and growth/reproductive traits (Figures 6 and 7, Table 2). This result may be explained by *A. thaliana's* status as a systemic endopolyploid which display endopolyploidy in the majority of their tissues (as opposed to localized endopolyploids that display endopolyploidy in the minority of their tissues) (De Rocher et al., 1990; Galbraith, Harkins, & Knapp, 1991; Yang & Loh, 2004). Systemic endopolyploidy in *A. thaliana* appears to be associated primarily with growth, as increasing endopolyploidy results in increased cell size and consequently organ size (Cookson, Radziejwoski, & Granier, 2006; Galbraith et al., 1991; Melaragno et al., 1993). Since *A. thaliana* already experiences extensive rounds of WGD (through endopolyploidy) during its development, introducing an extra round of organism‐level WGD (through induced polyploidy) could potentially be compensated for by systemically decreasing endopolyploidy (Figure 4a,b, Table 1, Appendix Table A1). This systemic compensation effect could then prevent any effects of induced polyploidy on plant functional traits, aside from the effects of increased base genome and cell size (Figures 4 and 5, Table 1), as well as potential effects of genome duplication on metabolic activity and gene regulation (Barow, 2006).

The experimental induction of polyploidy caused variable trait responses among accessions (Figures 4 and 5, Table 1). This variability was not caused by endopolyploidy which was shown by the lack of an interaction between cytotype and EI in our ANCOVA, suggesting that some other mechanism or mechanisms are responsible (Figures 6 and 7, Table 2). It is likely that multiple evolutionary mechanisms (i.e., natural selection, genetic drift or mutation) caused responses to induced polyploidy to diverge among accessions, but we are unable to determine which mechanism is most likely. If natural selection was responsible for the divergence among populations, accessions that show no trait response to polyploidy are more likely to persist after formation, since their function will not considerably differ from the evolutionary optimum of their progenitor population. By contrast, individuals from accessions where polyploidy caused major trait changes should be more likely to go extinct following formation. To determine whether natural selection was responsible for shaping the divergence of accession responses to autopolyploidy, large‐scale studies that introduce induced autopolyploids to their natural environments and compare their fitness to their diploid progenitors are needed. Despite a lack of information on the causes of variation in phenotypic responses to polyploidy, observing that phenotypic consequences of autopolyploidy are not uniform across the geographic range of a plant species can explain why the consequences of polyploidy on plant function and ecological distribution are often idiosyncratic (Otto & Whitton, 2000; Martin & Husband, 2009; Soltis et al., 2010; te Beest et al., 2012).

If induced polyploidy had less of an effect on plant functional traits than endopolyploidy, then why is polyploidy rare in natural populations of *A. thaliana* (del Pozo & Ramirez‐Parra, 2015; Weigel & Mott, 2009; Table 2)? One possible explanation is that polyploidy increases the number of days to bolting to a point that it is maladaptive in *A. thaliana*. Because *A. thaliana* is an annual with a short generation time, increasing the number of days it needs to complete its lifecycle may decrease its fitness in short‐lived ephemeral habitats (Galbraith et al., 1991; Otto & Whitton, 2000; Sherrard & Maherali, 2006; te Beest et al., 2012). That days to bolting was the functional trait that was most affected by induced polyploidy (increasing by 19%) is consistent with this explanation and may indicate that generation time in *A. thaliana* is particularly susceptible to changes in base genome size (Figure 4c, Table 1).

Our study provided novel insights about how induced polyploidy and endopolyploidy interact. It provides experimental evidence to support the hypothesis that inducing polyploidy decreases the degree of endopolyploidy (Figure 4a,b, Table 1, Appendix Table A1). Moreover, it shows that this trade‐off may be influenced by the genetic composition of populations, as only natural accessions (in contrast to laboratory‐based accessions used in prior experiments) of *A. thaliana* follow this pattern (del Pozo & Ramirez‐Parra, 2014; Rédei, 1992). Furthermore, we show that experimentally induced polyploidy does not affect the relationship between a trait and endopolyploidy which to our knowledge, has not been previously demonstrated (Figures 6 and 7, Table 2). Finally, our results highlight that phenotypic responses to autopolyploidy may not be easily predictable because of strong cytotype by accession interactions. Thus, the phenotypic consequences of genome duplication could vary across the geographic range of plant species.

## CONFLICT OF INTEREST

The authors have no conflicts of interest to declare.

## AUTHOR CONTRIBUTIONS

This study was equally designed by EKP, HM and BCH. EKP performed the research and collected and analyzed data. This manuscript was written primarily by EKP with significant contributions from HM and BCH.

## Data Availability

Dataset for individual endopolyploidy index, ploidy states and plant functional traits available at Dryad Digital Repository: https://doi.org/10.5061/dryad.280gb5mm9.

## APPENDIX 1

**Table A1:** Stock codes, geographic locations and average leaf and stem endopolyploidy index (EI) of diploid (2x) and tetraploid (4x) cytotypes of 55 accessions of *Arabidopsis thaliana*

Accession	Stock #	Latitude	Longitude	2x Leaf EI	2x Stem EI	4x Leaf EI	4x Stem EI
Ag‐0	CS22630	45.00	1.30	2.12	1.29	1.48	0.92
Aitba‐2	CS76347	31.48	−7.45	2.34	1.19	2.25	1.03
Angit‐1	CS76366	38.76	16.24	2.06	1.40	1.77	1.16
Bd‐0	CS962	52.46	13.29	1.74	1.30	1.51	1.08
Boot‐1	CS22551	54.40	−3.27	1.82	1.38	1.46	1.26
Bor‐4	CS22591	49.40	16.23	2.10	1.34	1.71	1.08
Borsk‐2	CS76421	53.04	51.75	2.33	1.23	1.99	0.98
Bozen‐1	CS76358	46.51	11.33	2.41	0.97	2.19	0.87
Castelfed‐4–212	CS76355	46.34	11.29	2.12	0.97	1.93	0.79
Chat‐1	CS22521	48.07	1.34	1.98	1.42	1.72	1.12
Ciste‐2	CS76360	41.62	12.87	2.03	1.14	1.89	0.91
Dr‐0	CS6684	51.05	13.73	1.92	1.27	1.71	1.02
Es‐0	CS1144	60.20	24.57	1.92	0.89	1.54	0.75
Fei‐0	CS76412	40.92	−8.54	1.82	1.34	1.60	1.20
Gd‐1	CS6716	53.50	10.50	2.06	1.40	1.67	1.23
Gel‐1	CS22533	51.02	5.87	1.98	1.29	1.66	1.06
Gr‐1	CS1198	47.00	15.50	2.05	1.27	1.72	1.00
HKT2‐4	CS76404	48.14	9.40	2.19	1.45	1.94	1.17
HR‐5	CS22596	51.41	−0.64	1.78	1.43	1.74	1.18
In‐0	CS1238	47.50	11.50	2.09	1.09	1.73	0.98
Istisu‐1	CS76389	38.98	48.56	2.20	1.30	1.95	0.89
Je‐0	CS6742	50.93	11.59	2.32	1.17	2.15	1.00
Ka‐0	CS6752	47.00	14.00	2.15	1.36	1.82	1.12
Koch‐1	CS76396	50.36	29.32	1.98	1.23	1.71	0.90
Kondara	CS22651	38.48	68.49	2.01	1.23	2.03	1.10
Lago‐1	CS76367	39.18	16.26	2.32	1.18	1.97	1.06
Lip‐0	CS1336	50.00	19.30	1.82	1.33	1.65	1.10
Lz‐0	CS22615	46.00	3.30	2.19	1.22	1.91	1.17
Mammo‐1	CS76365	38.36	16.23	1.94	1.29	1.73	0.96
Mer‐6	CS76414	38.92	−6.34	1.87	1.15	1.65	1.02
Ms‐0	CS22655	55.75	37.63	2.07	1.29	1.66	1.08
N‐13	CS22621	61.36	34.15	2.31	1.23	2.14	1.03
Nei1‐2	CS76402	48.52	8.80	1.80	1.24	1.61	1.02
Qui‐0	CS76417	42.69	−6.93	1.99	1.16	2.12	0.93
Rou‐0	CS6847	49.44	1.10	1.80	1.27	1.62	1.16
Rubezhnoe‐1	CS927	49.00	38.28	2.28	1.31	1.90	1.06
Sap‐0	CS1506	49.49	14.24	2.07	1.22	1.60	0.90
Sha	CS76382	37.29	71.30	1.97	1.44	1.62	1.29
Shigu‐2	CS76374	53.33	49.48	2.15	1.32	1.73	1.02
Sij‐1	CS76379	41.45	70.05	2.37	1.11	1.95	0.99
Sij‐2	CS76380	41.45	70.05	2.32	1.17	1.74	0.72
Sij‐4	CS76381	41.45	70.05	2.44	1.16	1.97	1.01
Slavi‐1	CS76419	41.43	23.65	2.19	1.19	1.50	0.95
Sorbo	CS22653	38.35	68.48	2.10	1.21	1.85	0.90
Stepn‐1	CS76378	54.06	60.48	2.11	1.22	1.78	1.05
Stw‐0	CS1538	52.00	36.00	2.01	1.60	1.71	1.21
Tu‐0	CS1566	45.00	7.50	2.15	1.37	1.88	1.18
Tue‐SB30‐3	CS76403	48.53	9.06	2.19	1.45	1.80	0.94
Tu‐Scha‐9	CS76401	48.53	9.05	1.88	1.25	1.69	1.10
Tu‐Wa1‐2	CS76405	48.53	9.03	1.69	1.04	1.42	0.90
Valsi‐1	CS76425	40.18	16.45	1.80	1.17	1.47	1.04
Voeran‐1	CS76352	46.36	11.23	2.37	1.17	1.97	1.03
Wal‐HasB‐4	CS76408	48.60	9.19	1.83	1.25	1.49	0.93
Ws‐2	CS22659	52.30	30.00	2.21	1.53	1.56	1.13
Yeg‐1	CS76394	39.87	45.36	2.29	1.31	1.61	1.11

Note

*N* = 2–3 for average leaf and stem EI, *N* = 1 for average 4x Sij‐1 stem EI.

**Table A2:** Average percentage of leaf cells within each ploidy state or that have experienced endopolyploidy (≥4C) in diploid (2x) and tetraploid (4x) cytotypes of 55 accessions of *Arabidopsis thaliana*

Accession	Cyto	2C (%)	4C (%)	8C (%)	16C (%)	32C (%)	64C (%)	128C (%)	256C (%)	512C (%)	1,024C (%)	2,048C (%)	≥4C (%)
Ag‐0	2x	12.02	18.66	23.61	37.61	7.15	0.81	0.11	0.02	0.02	0.00	0.00	87.98
Ag‐0	4x	21.80	24.85	38.35	13.99	0.92	0.04	0.00	0.06	0.00	0.00	0.00	78.20
Aitba‐2	2x	17.35	21.88	11.35	18.74	20.73	9.08	0.81	0.04	0.01	0.00	0.00	82.65
Aitba‐2	4x	14.90	16.92	20.09	28.98	15.36	3.48	0.19	0.08	0.00	0.00	0.00	85.10
Angit‐1	2x	11.65	20.30	24.96	37.33	5.21	0.46	0.10	0.00	0.00	0.00	0.00	88.35
Angit‐1	4x	15.15	21.51	37.85	22.84	2.34	0.26	0.03	0.01	0.00	0.00	0.00	84.85
Bd‐0	2x	15.56	25.96	29.55	27.37	1.30	0.23	0.01	0.01	0.00	0.00	0.00	84.44
Bd‐0	4x	18.15	27.23	42.67	10.60	0.94	0.17	0.03	0.10	0.11	0.00	0.00	81.85
Boot‐1	2x	13.18	24.07	32.40	28.42	1.73	0.16	0.03	0.00	0.00	0.00	0.00	86.82
Boot‐1	4x	18.38	31.06	38.53	10.97	0.86	0.13	0.00	0.06	0.00	0.00	0.00	81.62
Bor‐4	2x	12.50	21.21	20.87	35.72	8.94	0.72	0.04	0.00	0.00	0.00	0.00	87.50
Bor‐4	4x	13.63	23.16	43.74	17.81	1.42	0.17	0.00	0.05	0.02	0.00	0.00	86.37
Borsk‐2	2x	12.53	18.04	15.59	33.99	18.01	1.60	0.18	0.03	0.00	0.02	0.00	87.47
Borsk‐2	4x	17.90	21.14	21.45	24.59	13.59	1.28	0.02	0.03	0.00	0.00	0.00	82.10
Bozen‐1	2x	13.63	18.75	12.67	27.25	23.87	3.41	0.41	0.01	0.00	0.00	0.00	86.37
Bozen‐1	4x	18.29	15.40	18.90	26.32	19.01	1.91	0.05	0.12	0.02	0.00	0.00	81.71
Castelfed‐4‐212	2x	15.69	20.18	17.87	30.49	14.36	1.35	0.02	0.00	0.04	0.00	0.00	84.31
Castelfed‐4‐212	4x	18.05	18.74	27.33	25.02	9.49	1.29	0.07	0.00	0.00	0.00	0.00	81.95
Chat‐1	2x	10.86	23.48	24.57	38.72	2.21	0.12	0.01	0.00	0.01	0.00	0.00	89.14
Chat‐1	4x	17.90	20.49	35.86	24.38	0.76	0.11	0.11	0.21	0.16	0.00	0.00	82.10
Ciste‐2	2x	15.90	23.11	17.48	30.73	11.21	1.41	0.15	0.01	0.00	0.00	0.00	84.10
Ciste‐2	4x	17.08	18.40	30.56	27.50	5.43	0.85	0.07	0.07	0.03	0.00	0.00	82.92
Dr‐0	2x	11.78	21.72	31.07	33.44	1.85	0.11	0.01	0.00	0.01	0.01	0.00	88.22
Dr‐0	4x	13.58	23.40	42.91	19.30	0.70	0.05	0.04	0.02	0.00	0.00	0.00	86.42
Es‐0	2x	17.74	25.50	16.02	29.72	10.04	0.79	0.15	0.00	0.05	0.00	0.00	82.26
Es‐0	4x	28.77	22.77	20.09	23.28	4.24	0.72	0.07	0.06	0.00	0.00	0.00	71.23
Fei‐0	2x	11.13	21.82	42.10	23.55	1.27	0.09	0.03	0.00	0.00	0.00	0.00	88.87
Fei‐0	4x	14.90	24.30	47.17	12.99	0.57	0.07	0.01	0.00	0.00	0.00	0.00	85.10
Gd‐1	2x	10.32	20.16	26.95	39.97	2.08	0.17	0.02	0.03	0.16	0.16	0.00	89.68
Gd‐1	4x	14.94	24.96	40.08	18.73	1.00	0.12	0.01	0.17	0.00	0.00	0.00	85.06
Gel‐1	2x	12.07	23.23	24.18	36.94	3.20	0.09	0.05	0.02	0.14	0.08	0.00	87.93
Gel‐1	4x	17.65	23.18	36.55	21.40	1.14	0.08	0.00	0.00	0.01	0.00	0.00	82.35
Gr‐1	2x	13.37	20.49	23.97	32.32	9.41	0.37	0.00	0.03	0.03	0.02	0.00	86.63
Gr‐1	4x	15.74	23.35	35.98	23.15	1.71	0.06	0.02	0.00	0.00	0.00	0.00	84.26
HKT2‐4	2x	10.04	19.03	18.58	46.71	5.50	0.10	0.02	0.01	0.00	0.02	0.00	89.96
HKT2‐4	4x	13.52	20.88	27.71	34.21	3.46	0.09	0.04	0.06	0.02	0.00	0.00	86.48
HR‐5	2x	12.69	27.92	30.26	27.30	1.54	0.26	0.01	0.00	0.00	0.00	0.00	87.31
HR‐5	4x	14.43	23.19	38.79	21.81	1.52	0.11	0.04	0.05	0.07	0.00	0.00	85.57
In‐0	2x	15.59	19.63	16.78	36.56	10.81	0.61	0.02	0.00	0.00	0.00	0.00	84.41
In‐0	4x	18.88	23.62	28.26	24.86	3.91	0.43	0.02	0.00	0.02	0.00	0.00	81.12
Istisu‐1	2x	17.55	20.92	13.80	24.24	19.46	3.68	0.32	0.01	0.01	0.00	0.00	82.45
Istisu‐1	4x	19.04	21.08	23.43	21.78	12.42	1.90	0.25	0.10	0.00	0.00	0.00	80.96
Je‐0	2x	14.69	17.18	15.09	30.71	19.69	2.43	0.18	0.01	0.01	0.00	0.00	85.31
Je‐0	4x	13.21	14.99	26.88	34.37	9.78	0.77	0.00	0.00	0.00	0.00	0.00	86.79
Ka‐0	2x	10.93	18.84	20.99	44.32	4.29	0.44	0.03	0.02	0.03	0.08	0.03	89.07
Ka‐0	4x	10.51	23.17	42.29	22.03	1.83	0.16	0.02	0.00	0.00	0.00	0.00	89.49
Koch‐1	2x	11.85	22.26	25.85	36.02	3.74	0.25	0.02	0.00	0.00	0.01	0.00	88.15
Koch‐1	4x	18.99	20.94	33.50	23.74	2.42	0.29	0.06	0.06	0.00	0.00	0.00	81.01
Kondara	2x	12.14	21.24	27.81	31.69	6.59	0.44	0.07	0.02	0.00	0.00	0.00	87.86
Kondara	4x	14.99	19.41	24.17	30.76	10.07	0.47	0.08	0.05	0.00	0.00	0.00	85.01
Lago‐1	2x	9.36	20.86	15.11	40.08	12.35	2.04	0.16	0.01	0.00	0.03	0.00	90.64
Lago‐1	4x	18.35	19.86	22.37	26.88	10.87	1.66	0.02	0.00	0.00	0.00	0.00	81.65
Lip‐0	2x	12.45	23.28	36.87	24.45	2.68	0.20	0.05	0.01	0.00	0.00	0.00	87.55
Lip‐0	4x	14.83	24.86	42.27	16.44	1.42	0.12	0.01	0.04	0.01	0.00	0.00	85.17
Lz‐0	2x	11.92	16.14	19.98	45.39	6.24	0.30	0.01	0.01	0.00	0.00	0.00	88.08
Lz‐0	4x	11.99	20.84	34.62	30.10	2.19	0.22	0.01	0.01	0.00	0.01	0.00	88.01
Mammo‐1	2x	18.81	23.43	15.99	29.84	11.15	0.63	0.08	0.01	0.06	0.00	0.00	81.19
Mammo‐1	4x	20.46	22.97	24.90	26.76	4.33	0.48	0.04	0.06	0.00	0.00	0.00	79.54
Mer‐6	2x	13.89	20.03	33.60	29.94	2.45	0.07	0.00	0.01	0.00	0.00	0.00	86.11
Mer‐6	4x	16.44	21.78	43.97	16.10	1.63	0.08	0.00	0.00	0.00	0.00	0.00	83.56
Ms‐0	2x	11.97	18.93	25.28	38.11	5.23	0.45	0.03	0.00	0.00	0.00	0.00	88.03
Ms‐0	4x	16.40	23.31	39.74	19.04	1.29	0.22	0.00	0.00	0.00	0.00	0.00	83.60
N‐13	2x	13.83	20.18	14.74	27.78	19.73	3.41	0.29	0.00	0.03	0.00	0.00	86.17
N‐13	4x	14.53	23.52	19.36	25.16	12.23	4.25	0.88	0.08	0.00	0.00	0.00	85.47
Nei1‐2	2x	12.43	25.19	34.58	25.72	1.84	0.18	0.05	0.00	0.01	0.00	0.00	87.57
Nei1‐2	4x	15.53	25.43	42.50	15.43	0.95	0.14	0.00	0.01	0.00	0.00	0.00	84.47
Qui‐0	2x	12.86	20.73	23.25	40.87	2.06	0.20	0.03	0.00	0.00	0.00	0.00	87.14
Qui‐0	4x	10.16	16.27	29.92	39.22	4.11	0.26	0.02	0.03	0.00	0.00	0.00	89.84
Rou‐0	2x	9.97	23.69	45.21	19.11	1.70	0.27	0.04	0.00	0.01	0.00	0.00	90.03
Rou‐0	4x	13.32	26.47	46.92	11.66	1.38	0.21	0.02	0.00	0.02	0.00	0.00	86.68
Rubezhnoe‐1	2x	11.98	16.55	18.85	38.12	13.32	1.07	0.09	0.01	0.03	0.00	0.00	88.02
Rubezhnoe‐1	4x	16.22	18.58	29.53	31.12	4.03	0.39	0.10	0.03	0.00	0.00	0.00	83.78
Sap‐0	2x	9.17	19.95	29.59	37.61	3.38	0.21	0.02	0.02	0.03	0.01	0.01	90.83
Sap‐0	4x	11.95	26.14	52.88	8.29	0.69	0.03	0.01	0.00	0.00	0.00	0.00	88.05
Sha	2x	11.26	24.87	24.66	34.30	4.64	0.23	0.04	0.00	0.00	0.00	0.00	88.74
Sha	4x	18.99	22.69	37.12	20.14	0.87	0.14	0.01	0.03	0.01	0.00	0.00	81.01
Shigu‐2	2x	11.24	19.66	20.97	40.09	7.57	0.38	0.06	0.01	0.03	0.00	0.00	88.76
Shigu‐2	4x	16.23	23.22	34.39	23.83	2.02	0.22	0.06	0.04	0.00	0.00	0.00	83.77
Sij‐1	2x	10.94	17.62	16.60	34.66	18.71	1.33	0.09	0.01	0.04	0.00	0.00	89.06
Sij‐1	4x	14.25	20.07	28.95	30.73	5.27	0.62	0.04	0.06	0.00	0.00	0.00	85.75
Sij‐2	2x	11.10	15.97	19.50	37.88	14.78	0.65	0.09	0.03	0.00	0.00	0.00	88.90
Sij‐2	4x	17.60	23.59	29.83	25.60	3.07	0.29	0.03	0.00	0.00	0.00	0.00	82.40
Sij‐4	2x	15.22	16.26	10.88	28.06	26.55	2.83	0.13	0.05	0.00	0.00	0.00	84.78
Sij‐4	4x	17.70	17.98	21.73	35.20	7.08	0.30	0.00	0.00	0.00	0.00	0.00	82.30
Slavi‐1	2x	12.61	19.18	17.70	38.08	11.65	0.71	0.03	0.01	0.03	0.00	0.00	87.39
Slavi‐1	4x	29.67	20.00	23.98	23.30	2.88	0.14	0.02	0.02	0.00	0.00	0.00	70.33
Sorbo	2x	14.12	22.88	15.46	34.99	11.88	0.59	0.06	0.02	0.01	0.00	0.00	85.88
Sorbo	4x	19.98	21.10	21.49	30.00	6.86	0.42	0.11	0.03	0.00	0.00	0.00	80.02
Stepn‐1	2x	13.80	19.18	19.95	37.26	8.99	0.65	0.11	0.06	0.00	0.00	0.00	86.20
Stepn‐1	4x	14.43	22.11	37.85	23.07	2.25	0.23	0.03	0.00	0.02	0.00	0.00	85.57
Stw‐0	2x	11.03	21.05	26.70	38.33	2.70	0.16	0.03	0.00	0.00	0.00	0.00	88.97
Stw‐0	4x	10.37	23.89	50.56	14.43	0.60	0.14	0.00	0.00	0.00	0.00	0.00	89.63
Tu‐0	2x	10.57	18.25	21.89	44.41	4.67	0.18	0.00	0.02	0.01	0.00	0.00	89.43
Tu‐0	4x	11.28	20.36	38.85	28.14	1.19	0.09	0.06	0.01	0.02	0.00	0.00	88.72
Tue‐SB30‐3	2x	11.63	19.27	18.95	39.31	10.50	0.31	0.01	0.01	0.00	0.00	0.00	88.37
Tue‐SB30‐3	4x	14.90	21.15	35.70	26.08	2.08	0.07	0.03	0.00	0.00	0.00	0.00	85.10
Tu‐Scha‐9	2x	11.53	21.23	22.79	35.97	7.87	0.54	0.05	0.00	0.01	0.00	0.00	88.47
Tu‐Scha‐9	4x	14.45	22.03	45.06	16.99	1.28	0.18	0.00	0.00	0.00	0.00	0.00	85.55
Tu‐Wa1‐2	2x	10.91	26.57	46.51	14.89	0.82	0.27	0.02	0.01	0.00	0.00	0.00	89.09
Tu‐Wa1‐2	4x	15.22	35.22	42.77	6.26	0.43	0.08	0.00	0.01	0.00	0.00	0.00	84.78
Valsi‐1	2x	19.19	22.30	23.50	30.60	3.67	0.67	0.06	0.02	0.00	0.00	0.00	80.81
Valsi‐1	4x	28.63	19.90	30.84	17.35	2.93	0.35	0.00	0.00	0.00	0.00	0.00	71.37
Voeran‐1	2x	13.92	18.47	12.39	30.03	22.74	2.10	0.30	0.03	0.02	0.00	0.00	86.08
Voeran‐1	4x	16.42	20.73	22.53	30.92	8.62	0.77	0.02	0.00	0.00	0.00	0.00	83.58
Wal‐HasB‐4	2x	10.25	21.53	44.88	22.22	1.04	0.07	0.01	0.00	0.00	0.00	0.00	89.75
Wal‐HasB‐4	4x	14.81	29.54	48.19	6.87	0.43	0.06	0.00	0.01	0.09	0.00	0.00	85.19
Ws‐2	2x	9.79	17.68	21.10	44.74	6.40	0.27	0.02	0.00	0.00	0.00	0.00	90.21
Ws‐2	4x	14.05	31.26	40.73	13.11	0.77	0.06	0.00	0.02	0.00	0.00	0.00	85.95
Yeg‐1	2x	9.23	18.14	18.35	43.34	10.26	0.61	0.01	0.02	0.02	0.01	0.00	90.77
Yeg‐1	4x	18.10	24.64	39.06	15.21	2.52	0.45	0.02	0.00	0.00	0.00	0.00	81.90

Note

The 2C peak of a diploid is 2x (4C = 4x, 8C = 8x etc.) while the 2C peak of a tetraploid is 4x (4C = 8x, 8C = 16x etc.). *N* = 2–3 for each specific accession and cytotype.

**Table A3:** Average percentage of stem cells within each ploidy state or that have experienced endopolyploidy (≥4C) in diploid (2x) and tetraploid (4x) cytotypes of 55 accessions of *Arabidopsis thaliana*

Accession	Cyto	2C (%)	4C (%)	8C (%)	16C (%)	32C (%)	64C (%)	128C (%)	256C (%)	512C (%)	1,024C (%)	2,048C (%)	≥4C (%)
Ag‐0	2x	13.61	52.55	26.06	7.19	0.40	0.18	0.01	0.00	0.00	0.00	0.00	86.39
Ag‐0	4x	26.48	56.77	15.22	1.38	0.13	0.02	0.00	0.00	0.00	0.00	0.00	73.52
Aitba‐2	2x	20.60	48.65	22.79	7.54	0.33	0.10	0.00	0.00	0.00	0.00	0.00	79.40
Aitba‐2	4x	26.14	50.76	17.21	5.53	0.25	0.11	0.00	0.00	0.00	0.00	0.00	73.86
Angit‐1	2x	19.37	41.49	21.87	14.97	2.05	0.20	0.04	0.00	0.00	0.00	0.00	80.63
Angit‐1	4x	22.95	48.23	20.11	7.85	0.72	0.14	0.00	0.00	0.00	0.00	0.00	77.05
Bd‐0	2x	23.36	38.80	23.81	12.59	1.16	0.25	0.04	0.00	0.00	0.00	0.00	76.64
Bd‐0	4x	28.82	44.37	18.14	7.71	0.89	0.03	0.03	0.00	0.00	0.00	0.00	71.18
Boot‐1	2x	17.18	45.32	22.36	13.32	1.63	0.15	0.04	0.00	0.00	0.00	0.00	82.82
Boot‐1	4x	20.32	45.02	24.03	9.77	0.61	0.24	0.02	0.00	0.00	0.00	0.00	79.68
Bor‐4	2x	17.12	45.97	24.59	10.98	0.99	0.30	0.04	0.01	0.00	0.00	0.00	82.88
Bor‐4	4x	23.69	50.19	21.68	3.44	0.73	0.20	0.06	0.00	0.00	0.00	0.00	76.31
Borsk‐2	2x	20.99	48.65	18.73	9.85	1.54	0.14	0.06	0.02	0.02	0.00	0.00	79.01
Borsk‐2	4x	30.29	47.59	16.79	4.97	0.31	0.05	0.00	0.00	0.00	0.00	0.00	69.71
Bozen‐1	2x	25.84	53.81	17.95	2.20	0.11	0.10	0.00	0.00	0.00	0.00	0.00	74.16
Bozen‐1	4x	34.87	46.58	15.55	2.67	0.30	0.02	0.00	0.00	0.00	0.00	0.00	65.13
Castelfed‐4‐212	2x	26.17	54.07	16.77	2.41	0.49	0.09	0.00	0.00	0.00	0.00	0.00	73.83
Castelfed‐4‐212	4x	36.03	51.43	10.71	1.58	0.17	0.04	0.04	0.00	0.00	0.00	0.00	63.97
Chat‐1	2x	18.74	37.11	28.68	14.56	0.69	0.19	0.02	0.00	0.00	0.00	0.00	81.26
Chat‐1	4x	25.45	42.63	26.63	4.93	0.33	0.03	0.00	0.00	0.00	0.00	0.00	74.55
Ciste‐2	2x	19.85	53.23	21.55	4.49	0.51	0.24	0.13	0.00	0.00	0.00	0.00	80.15
Ciste‐2	4x	31.47	48.59	17.80	2.04	0.11	0.00	0.00	0.00	0.00	0.00	0.00	68.53
Dr‐0	2x	18.67	49.90	18.93	11.13	1.15	0.16	0.06	0.00	0.00	0.00	0.00	81.33
Dr‐0	4x	29.20	46.97	17.54	5.77	0.42	0.09	0.00	0.00	0.00	0.00	0.00	70.80
Es‐0	2x	28.56	56.71	12.48	1.89	0.26	0.07	0.03	0.00	0.00	0.00	0.00	71.44
Es‐0	4x	38.93	48.88	10.84	1.17	0.11	0.06	0.00	0.00	0.00	0.00	0.00	61.07
Fei‐0	2x	17.75	46.23	22.17	12.33	1.22	0.29	0.00	0.00	0.00	0.00	0.00	82.25
Fei‐0	4x	22.31	44.38	25.03	7.86	0.36	0.05	0.00	0.00	0.00	0.00	0.00	77.69
Gd‐1	2x	16.81	45.70	21.49	12.55	3.27	0.16	0.02	0.00	0.00	0.00	0.00	83.19
Gd‐1	4x	23.08	45.44	19.74	9.30	2.31	0.11	0.01	0.00	0.00	0.00	0.00	76.92
Gel‐1	2x	18.45	49.54	18.64	11.43	1.77	0.14	0.02	0.00	0.00	0.00	0.00	81.55
Gel‐1	4x	28.56	44.66	19.58	6.66	0.45	0.06	0.00	0.00	0.02	0.00	0.00	71.44
Gr‐1	2x	21.22	42.52	25.29	9.87	0.92	0.16	0.01	0.00	0.00	0.00	0.00	78.78
Gr‐1	4x	26.43	50.64	19.56	2.97	0.40	0.00	0.00	0.00	0.00	0.00	0.00	73.57
HKT2‐4	2x	16.06	39.52	28.66	14.99	0.71	0.05	0.01	0.00	0.00	0.00	0.00	83.94
HKT2‐4	4x	22.43	44.13	28.02	4.96	0.46	0.00	0.00	0.00	0.00	0.00	0.00	77.57
HR‐5	2x	17.36	41.45	23.52	16.25	1.16	0.22	0.04	0.00	0.00	0.00	0.00	82.64
HR‐5	4x	24.67	42.32	24.03	8.69	0.22	0.07	0.00	0.00	0.00	0.00	0.00	75.33
In‐0	2x	27.65	46.20	17.04	8.26	0.77	0.08	0.00	0.00	0.00	0.00	0.00	72.35
In‐0	4x	28.99	49.19	16.68	4.78	0.34	0.02	0.00	0.00	0.00	0.00	0.00	71.01
Istisu‐1	2x	17.56	49.86	19.74	11.41	1.24	0.13	0.04	0.02	0.00	0.00	0.00	82.44
Istisu‐1	4x	34.33	46.35	15.91	3.02	0.38	0.02	0.00	0.00	0.00	0.00	0.00	65.67
Je‐0	2x	19.78	51.10	22.27	6.17	0.56	0.05	0.07	0.00	0.00	0.00	0.00	80.22
Je‐0	4x	28.79	47.57	18.87	4.44	0.25	0.08	0.00	0.00	0.00	0.00	0.00	71.21
Ka‐0	2x	18.65	44.49	20.92	14.37	1.45	0.11	0.02	0.00	0.00	0.00	0.00	81.35
Ka‐0	4x	23.17	47.78	22.85	5.82	0.36	0.00	0.02	0.00	0.00	0.00	0.00	76.83
Koch‐1	2x	18.27	52.00	20.12	8.45	0.84	0.26	0.06	0.00	0.00	0.00	0.00	81.73
Koch‐1	4x	30.74	51.57	14.87	2.40	0.34	0.07	0.01	0.00	0.00	0.00	0.00	69.26
Kondara	2x	20.83	47.71	20.81	9.46	0.76	0.39	0.03	0.01	0.00	0.00	0.00	79.17
Kondara	4x	24.19	48.25	21.39	5.56	0.58	0.02	0.00	0.02	0.00	0.00	0.00	75.81
Lago‐1	2x	17.65	53.95	21.82	5.99	0.46	0.05	0.08	0.00	0.00	0.00	0.00	82.35
Lago‐1	4x	26.52	48.14	19.56	4.93	0.63	0.19	0.02	0.00	0.01	0.00	0.00	73.48
Lip‐0	2x	20.05	43.76	22.02	12.22	1.66	0.22	0.05	0.02	0.00	0.00	0.00	79.95
Lip‐0	4x	27.16	44.33	21.31	6.28	0.81	0.10	0.00	0.00	0.00	0.00	0.00	72.84
Lz‐0	2x	20.23	49.08	19.95	9.62	1.02	0.07	0.03	0.00	0.00	0.00	0.00	79.77
Lz‐0	4x	23.30	47.47	19.49	8.58	0.94	0.19	0.02	0.00	0.00	0.00	0.00	76.70
Mammo‐1	2x	17.71	47.07	25.62	8.57	0.61	0.33	0.08	0.00	0.00	0.00	0.00	82.29
Mammo‐1	4x	27.95	50.22	19.58	2.12	0.13	0.00	0.00	0.00	0.00	0.00	0.00	72.05
Mer‐6	2x	24.42	44.99	22.84	6.64	0.76	0.34	0.00	0.00	0.00	0.00	0.00	75.58
Mer‐6	4x	29.77	44.93	19.44	5.30	0.48	0.08	0.00	0.00	0.00	0.00	0.00	70.23
Ms‐0	2x	18.15	46.21	25.04	9.77	0.61	0.13	0.08	0.02	0.00	0.00	0.00	81.85
Ms‐0	4x	25.28	47.58	21.71	5.04	0.29	0.09	0.00	0.00	0.00	0.00	0.00	74.72
N‐13	2x	17.98	51.56	21.53	7.44	1.14	0.31	0.03	0.02	0.00	0.00	0.00	82.02
N‐13	4x	25.88	50.45	19.28	3.89	0.47	0.03	0.00	0.00	0.00	0.00	0.00	74.12
Nei1‐2	2x	22.41	44.50	20.90	11.24	0.91	0.04	0.00	0.00	0.00	0.00	0.00	77.59
Nei1‐2	4x	29.67	44.24	21.30	4.48	0.25	0.05	0.00	0.00	0.00	0.00	0.00	70.33
Qui‐0	2x	23.21	48.80	18.55	8.44	0.72	0.23	0.04	0.00	0.00	0.00	0.00	76.79
Qui‐0	4x	29.92	50.93	15.77	3.21	0.13	0.03	0.00	0.00	0.00	0.00	0.00	70.08
Rou‐0	2x	20.69	47.50	18.43	11.63	1.30	0.31	0.14	0.00	0.00	0.00	0.00	79.31
Rou‐0	4x	23.63	47.26	19.84	8.41	0.77	0.08	0.02	0.00	0.00	0.00	0.00	76.37
Rubezhnoe‐1	2x	20.55	43.05	23.68	10.90	1.53	0.22	0.03	0.03	0.00	0.00	0.00	79.45
Rubezhnoe‐1	4x	26.78	46.41	21.45	4.72	0.50	0.10	0.02	0.02	0.00	0.00	0.00	73.22
Sap‐0	2x	25.18	42.58	19.62	10.83	1.55	0.22	0.00	0.00	0.02	0.00	0.00	74.82
Sap‐0	4x	36.35	42.56	16.64	4.01	0.35	0.06	0.02	0.00	0.00	0.00	0.00	63.65
Sha	2x	13.98	46.21	23.91	13.97	1.72	0.18	0.03	0.00	0.00	0.00	0.00	86.02
Sha	4x	18.06	44.67	28.17	8.30	0.76	0.04	0.00	0.00	0.00	0.00	0.00	81.94
Shigu‐2	2x	17.34	48.75	21.35	10.20	2.09	0.25	0.03	0.00	0.00	0.00	0.00	82.66
Shigu‐2	4x	27.46	50.35	16.12	5.09	0.81	0.17	0.00	0.00	0.00	0.00	0.00	72.54
Sij‐1	2x	24.00	50.93	16.69	7.38	0.87	0.11	0.00	0.01	0.00	0.00	0.00	76.00
Sij‐1	4x	27.58	49.98	17.74	4.36	0.30	0.04	0.00	0.00	0.00	0.00	0.00	72.42
Sij‐2	2x	20.57	51.43	19.92	6.72	1.25	0.10	0.01	0.00	0.00	0.00	0.00	79.43
Sij‐2	4x	39.58	50.21	8.98	1.06	0.15	0.01	0.00	0.00	0.00	0.00	0.00	60.42
Sij‐4	2x	25.52	45.71	17.88	9.34	1.43	0.13	0.00	0.00	0.00	0.00	0.00	74.48
Sij‐4	4x	28.25	47.89	19.36	4.00	0.44	0.05	0.00	0.00	0.00	0.00	0.00	71.75
Slavi‐1	2x	23.78	45.35	20.18	9.73	0.83	0.15	0.00	0.00	0.00	0.00	0.00	76.22
Slavi‐1	4x	30.91	46.87	19.07	2.93	0.18	0.04	0.00	0.00	0.00	0.00	0.00	69.09
Sorbo	2x	22.89	46.68	18.81	10.14	1.36	0.13	0.00	0.00	0.00	0.00	0.00	77.11
Sorbo	4x	32.69	48.65	14.77	3.59	0.25	0.02	0.00	0.00	0.02	0.00	0.00	67.31
Stepn‐1	2x	18.48	51.38	21.71	7.34	0.69	0.32	0.08	0.00	0.00	0.00	0.00	81.52
Stepn‐1	4x	27.27	46.63	20.65	4.78	0.56	0.10	0.02	0.00	0.00	0.00	0.00	72.73
Stw‐0	2x	13.56	39.40	24.42	18.85	3.53	0.22	0.02	0.00	0.00	0.00	0.00	86.44
Stw‐0	4x	22.99	45.31	21.23	9.22	1.19	0.05	0.02	0.00	0.00	0.00	0.00	77.01
Tu‐0	2x	18.28	45.67	19.00	14.97	1.73	0.21	0.10	0.02	0.00	0.00	0.00	81.72
Tu‐0	4x	24.94	44.14	20.14	9.54	1.05	0.13	0.04	0.00	0.00	0.00	0.00	75.06
Tue‐SB30‐3	2x	15.10	45.17	23.09	13.27	3.02	0.31	0.05	0.00	0.00	0.00	0.00	84.90
Tue‐SB30‐3	4x	33.20	44.44	17.84	4.13	0.35	0.04	0.00	0.00	0.00	0.00	0.00	66.80
Tu‐Scha‐9	2x	14.61	39.56	31.02	12.47	1.72	0.47	0.16	0.00	0.00	0.00	0.00	85.39
Tu‐Scha‐9	4x	18.34	57.28	20.87	3.08	0.37	0.05	0.03	0.00	0.00	0.00	0.00	81.66
Tu‐Wa1‐2	2x	31.45	41.05	19.91	7.06	0.36	0.12	0.02	0.01	0.00	0.01	0.00	68.55
Tu‐Wa1‐2	4x	34.84	43.74	18.38	2.77	0.19	0.07	0.02	0.00	0.00	0.00	0.00	65.16
Valsi‐1	2x	19.18	53.34	19.76	6.72	0.75	0.23	0.02	0.00	0.00	0.00	0.00	80.82
Valsi‐1	4x	25.25	50.24	20.54	3.60	0.28	0.09	0.00	0.00	0.00	0.00	0.00	74.75
Voeran‐1	2x	19.37	51.15	22.96	5.76	0.66	0.08	0.02	0.00	0.00	0.00	0.00	80.63
Voeran‐1	4x	24.20	53.25	18.30	3.80	0.36	0.10	0.00	0.00	0.00	0.00	0.00	75.80
Wal‐HasB‐4	2x	20.71	44.88	24.84	8.73	0.47	0.32	0.05	0.00	0.00	0.00	0.00	79.29
Wal‐HasB‐4	4x	33.11	44.97	18.05	3.44	0.37	0.04	0.00	0.02	0.00	0.00	0.00	66.89
Ws‐2	2x	15.53	39.35	26.26	14.50	4.14	0.21	0.02	0.00	0.00	0.00	0.00	84.47
Ws‐2	4x	28.44	41.75	20.20	8.18	1.34	0.05	0.02	0.00	0.02	0.00	0.00	71.56
Yeg‐1	2x	20.73	46.16	17.98	12.18	2.61	0.30	0.03	0.00	0.00	0.00	0.00	79.27
Yeg‐1	4x	25.84	47.62	17.06	8.81	0.44	0.22	0.02	0.00	0.00	0.00	0.00	74.16

Note

The 2C peak of a diploid is 2x (4C = 4x, 8C = 8x etc.) while the 2C peak of a tetraploid is 4x (4C = 8x, 8C = 16x etc.). *N* = 2–3 for each specific accession and cytotype, *N* = 1 for 4x Sij‐1.
